# An examination of the association between marital status and prenatal mental disorders using linked health administrative data

**DOI:** 10.1186/s12884-022-05045-8

**Published:** 2022-10-01

**Authors:** Asres Bedaso, Jon Adams, Wenbo Peng, Fenglian Xu, David Sibbritt

**Affiliations:** 1 College of Medicine and Health Sciences, School of Nursing, Hawassa University, Hawassa, Ethiopia; 2 Australian Centre for Public and Population Health Research, School of Public Health, Faculty of Health, University of Technology Sydney, Ultimo, NSW Australia; 3 Data Analysis & Surgical Outcomes Unit, Royal North Shore Hospital, Sydney, NSW Australia

**Keywords:** Depressive disorder, Anxiety disorder, Self-harm, Marital status, Pregnancy, Data linkage

## Abstract

**Background:**

International research shows marital status impacts the mental health of pregnant women, with prenatal depression and anxiety being higher among non-partnered women. However, there have been few studies examining the relationship between marital status and prenatal mental disorders among Australian women.

**Methods:**

This is a population-based retrospective cohort study using linked data from the New South Wales (NSW) Perinatal Data Collection (PDC) and Admitted Patients Data Collection (APDC). The cohort consists of a total of 598,599 pregnant women with 865,349 admissions. Identification of pregnant women for mental disorders was conducted using the 10^th^ version International Classification of Diseases and Related Health Problems, Australian Modification (ICD-10-AM). A binary logistic regression model was used to estimate the relationship between marital status and prenatal mental disorder after adjusting for confounders.

**Results:**

Of the included pregnant women, 241 (0.04%), 107 (0.02%) and 4359 (0.5%) were diagnosed with depressive disorder, anxiety disorder, and self-harm, respectively. Non-partnered pregnant women had a higher likelihood of depressive disorder (Adjusted Odds Ratio (AOR) = 2.75; 95% CI: 2.04, 3.70) and anxiety disorder (AOR = 3.16, 95% CI: 2.03, 4.91), compared with partnered women. Furthermore, the likelihood of experiencing self-harm was two times higher among non-partnered pregnant women (AOR = 2.00; 95% CI: 1.82, 2.20) than partnered pregnant women.

**Conclusions:**

Non-partnered marital status has a significant positive association with prenatal depressive disorder, anxiety disorder and self-harm. This suggests it would be highly beneficial for maternal health care professionals to screen non-partnered pregnant women for prenatal mental health problems such as depression, anxiety and self-harm.

**Supplementary Information:**

The online version contains supplementary material available at 10.1186/s12884-022-05045-8.

## Background

Depression [1], anxiety [2] and self-harm [3] are among the most prevalent mental health problems during pregnancy. An international umbrella review indicated that the pooled prevalence of antenatal depression was 17% in high-income countries [4], while studies conducted in Australia have reported a prevalence of depression ranging from 7–17% [5, 6]. Also, various epidemiological studies have reported the prevalence of anxiety during pregnancy ranging from 14–59% [7–10], while a study conducted in Australia have reported a 27% prevalence of antenatal anxiety [5]. These prenatal mental health problems adversely impact the mother's physical and emotional well-being [11, 12] as well as the well-being of infants and children [13]. The 2019 Australian health-economic analysis of the impact of depression and anxiety during the perinatal period estimated the costs at $877 million in the first year [14]. The 2020 Productivity Commission Mental Health Report also estimated the cost of improving perinatal mental health at an additional $18–23 million in direct expenditure [15].

Antenatal depression and anxiety can result in adverse obstetric and foetal outcomes [11, 13, 16–19], and impaired mother-infant interaction [20–23]. In addition, prenatal mental health problems have a significant association with substance use thereby potentially resulting in impaired quality of life [2, 24, 25]. Based on reports from published studies, correlates of prenatal anxiety include pregnancy loss [26–28], physical abuse [29–31], history of mental illness [30–34], substance abuse [27, 30, 35, 36], unplanned pregnancy [37], and low social support [38]. Further, antenatal depression has a significant association with low social support, exposure to stressful events, low income, history of abuse [5, 39, 40], unplanned pregnancy, and history of any mental illness [41, 42].

Epidemiological studies show pregnant women with marital disruption or unmarried have a higher rate of developing prenatal depression [33, 43, 44] and anxiety [33] compared to partnered women. Also, a study conducted in Brazil demonstrated that the likelihood of prenatal suicide was significantly related to lack of a cohabiting partner [45]. Conversely, some studies conducted in Italy [46], UK [47] and US [48] report a non-significant association between marital status and prenatal depression. Further, a study conducted in China reported a non-significant association between marital status and prenatal anxiety [49].

Nonetheless, partnered pregnant women living in poor-quality relationships with their partners also appear to be at greater risk of prenatal mental health problems [38]. Also, a study conducted in Victoria, Australia found single mothers report higher levels of prenatal depressive symptoms than those with unsupportive partners [50]. A review of longitudinal studies conducted in Australia and New Zealand indicated poor partner relationship as the strongest predictor of prenatal anxiety and depression [34]. Also, pregnant women in a violent marital relationship receive less support from their spouses and even cause additional stress and anxiety leading to adverse birth outcomes, including low birth weight and preterm birth [51, 52].

The relationship between marital status and the risk of mental disorders, including depressive disorder, anxiety disorder and self-harm, has received little research attention in Australia and globally. There has also been a lack of studies utilising high quality linked data on this topic, which would create opportunities for more complex and expanded research. Also, it is vital to examine whether non-partnered status poses a particular disadvantage to pregnant women’s mental health in terms of depressive disorder, anxiety disorder and self-harm risk. Understanding such relationships is important to inform approaches for supporting non-partnered pregnant women with a view to enhancing their mental wellbeing.

In direct response to these gaps in the current literature, our large cohort study aimed to assess the association between marital status and prenatal mental disorders among Australian women using linked health administrative data from the State of New South Wales (NSW), Australia. We hypothesized that non-partnered marital status is significantly associated with prenatal depressive disorder, anxiety disorder and self-harm.

## Methods

### Data source and study population

The current population-based retrospective cohort study used linked data from the NSW Admitted Patients Data Collection (APDC) and the NSW Perinatal Data Collection (PDC) (https://www.cherel.org.au/data-dictionaries) and reported per the guideline of the STROBE checklist (Additional file 1). The APDC contains regularly collected data on inpatient services from all public and private hospitals, and public multi-purpose service centres in NSW. APDC contains the demographic characteristics, clinical diagnosis and other clinical procedures of the patient. The PDC collects data on pregnancy and all births (i.e., hospital and homebirths) of ≥ 20 weeks gestation or birth weight of ≥ 400 g.

### Eligibility criteria and sample selection

**Fig. 1:**
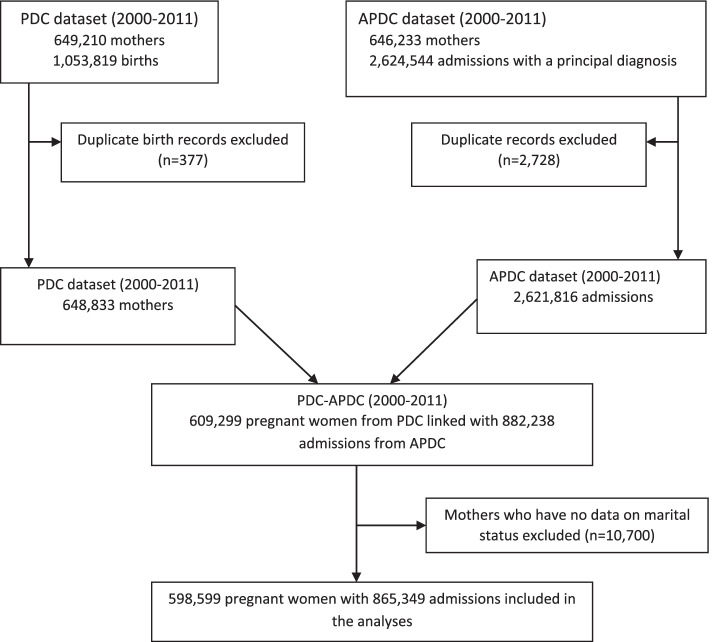
Study population and data linkage between PDC and APDC

The eligible criteria for inclusion in the current study were; first, women should be pregnant between 2000 and 2011 and resides in NSW. Second, there should be a report on their marital status. Hospital admissions for mental disorders were identified using data from the APDC dataset (2000–2011), which contain 646,233 mothers with 2,624,544 hospital admissions. In the PDC dataset (2000–2011), there were 649,210 mothers and 1,053,819 births. After excluding duplicate records 609,299 mothers from the PDC dataset were linked with 882,238 admissions from APDC dataset. After excluding those women who have no data on marital status, 598,599 pregnant women with 865,349 admissions included in the analyses. The details of the study population selection process and data linkage are presented in Fig. 1.

The Centre for Health Record Linkage (CHeReL) executed the data linkage between the PDC and APDC dataset using probabilistic record linkage methods and Choice-maker software for these two data sources [53], which estimated a false positive rate of 0.3% and a false negative < 0.5% of records.

### Diagnoses of mental disorders

The diagnosis of mental health disorders of pregnant women was performed by a doctor at the inpatient department of hospitals in NSW and coded per the criteria of the 10^th^ version International Classification of Diseases and Related Health Problems, Australian Modification (ICD-10-AM) [54]. ICD-10-AM is a diagnostic mental health measure that likely picks up the more severe mental health disorder within the population and misses women with milder mental health disorders compared with screening or self-report measures. The record admission contains one principal diagnosis, one stay diagnosis and 53 other diagnoses [55]. For the current study, only a principal diagnosis of depressive disorder, anxiety disorder or self-harm was considered for participant inclusion. The data for admission was taken from the Admitted Patients Data Collection (APDC) data set. A hospital admission refers to any hospitalisation or admission of a patient to hospital for inpatient service.

If a woman had the principal diagnosis of depressive episode [F32], recurrent depressive disorder [F33], persistent mood (affective) disorder [F34], other mood (affective) disorder [F38], or unspecified mood (affective) disorder [39], her admission was identified as depressive disorder. If a woman had the principal diagnosis of phobic anxiety disorders [F40], other anxiety disorders [F41], or obsessive–compulsive disorder [F42], her admission was identified as anxiety disorder. If a woman had the principal diagnosis of self-injuries and/or self-poisoning [S00-T75] or certain early complications of trauma [T79], her admission was identified as international self-harm.

### Exposure and confounding variables

The exposure variable was marital status of pregnant women. The available response options on the marital status were never married, married/de facto relationship, separated, divorced, or widowed. Then, those with a marital status of never married, separated, divorced, and widowed were categorised as “non-partnered”, whilst the remaining response (married and de facto relationship) were grouped as “partnered”. The confounding variables included in the analyses were age, remoteness, socio-economic disadvantage, country of birth, indigenous status, and smoking status.

### Statistical analysis

We undertook three steps to analyse the data. First, frequency and percentages were generated to show the prevalence of the outcome variables (depressive disorder, anxiety disorder and self-harm) among admitted pregnant were determined. Then, we cross-tabulated the distribution of the exposure variable (partnered or non-partnered) across the confounding and outcome variables; including using the Pearson’s chi-square test and Student’s t-test to demonstrate statistically significant associations. Further, a binary logistic regression model was employed to determine the association between marital status and mental disorders (i.e. depressive disorder, anxiety disorder and self-harm) after adjusting for confounders. The results of the regression analyses in the final model were described using adjusted odds ratio (AOR) with the respective 95% confidence intervals (CI). The final model was assessed using the Hosmer and Lemeshow goodness of fit test [56]. For all statistical analyses, statistical significance was set at *p* < 0.05. Analyses was conducted using STATA/MP 16 (Stata Corp, USA).

## Results

Among the 609,299 pregnant women who were living in NSW and their babies were firstborn between 2000 and 2011 by linking the APDC dataset and PDC dataset, 1.8% (*n* = 10,700) did not report their marital status. Pregnant women with missing data on this exposure variable (i.e. marital status) were excluded from the analyses and thus data from 598,599 pregnant women were included in the analyses. A total of 241 pregnant women (0.04%) were with the principal diagnosis of depressive disorder; 107 (0.02%) with the principal diagnosis of anxiety disorder; and 4,359 (0.5%) with the principal diagnosis of intentional self-harm. None of the pregnant women were found to experience two or all of these mental health conditions.

The socio-demographic characteristics of study participants are presented in Table 1. The average age of the pregnant women was 29.6 (SD = 5.8) years, with 31.8% (*n* = 193,439) of the women were between the age of 30–34 years and 28.1% (*n* = 171, 373) were aged 25–29 years. The majority of the pregnant women (67.4%) resided in a major city.

**Table 1:** Characteristics of study participants stratified by marital status

Factors	Marital status			
	**Non-partnered** (*n* = 112,443 women)	**Partnered** (*n* = 486,156 women)	**Total** (598,599 women)	
	**Mean (SD)**	**Mean (SD)**	**Mean (SD)**	***p*-value **^**A**^
**Age (mean ± SD)**	26.0 ± 6.6	30.4 ± 5.2	29.6 ± 5.8	< 0.001
	**n (%)**	**n (%)**	**n (%)**	***p*-value **^**B**^
**Remoteness**				
Major cities	63,309 (57.2)	335,633 (69.8)	398,942 (67.44)	< 0.001
Inner regional	33,119 (29.9)	107,812 (22.4)	140,931 (23.83)	
Out regional and remote	14,321 (12.9)	37,328 (7.8)	51,649 (8.73)	
**Socio-economic disadvantage**				
1^st^ quintile (least disadvantaged)	12,112 (10.9)	110,531 (23.0)	122,643 (20.73)	< 0.001
2^nd^ quintile	17,515 (15.8)	100,915 (21.0)	118,430 (20.02)	
3^rd^ quintile	21,867 (19.7)	89,191 (18.6)	111,058 (18.77)	
4^th^ quintile	27,937 (25.2)	82,829 (17.2)	110,766 (18.73)	
5^th^ quintile (most disadvantaged)	31,318 (28.3)	97,307 (20.2)	128,625 (21.74)	
**Smoking status**				
No	68,158 (65.5)	407,654 (91.3)	475,812 (86.45)	< 0.001
Yes	35,839 (34.5)	38,766 (8.7)	74,605 (18.38)	
**Country of birth**				
Australia	92,340 (82.1)	313,478 (64.5)	405,818 (67.79)	< 0.001
Other countries	20,103 (17.9)	172,678 (35.5)	192,781 (33.21)	
**Indigenous status**				
No	103,010 (92.3)	477,410 (98.9)	580,420 (97.67)	< 0.001
Yes	8,632 (7.7)	5,212 (1.1)	13,844 (4.54)	
**Insurance status**				
No cover	77,104 (88.4)	228,032 (56.6)	305,136 (62.26)	< 0.001
Basic cover	3,352 (3.8)	44,845 (11.1)	48,197 (9.83)	
Full cover	6,542 (7.5)	128,679 (31.9)	135,221 (27.59)	
Ancillary cover only	199 (0.2)	1,346 (0.3)	1,545 (0.32)	
**Depressive disorders**				
No/not stated	112,336 (99.9)	486,022 (99.9)	598,358 (99.96)	< 0.001
Yes	107 (0.1)	134 (0.03)	241 (0.04)	
**Anxiety disorder**				
No/not stated	112,400 (99.9)	486,092 (99.9)	598,492 (99.98)	< 0.001
Yes	43 (0.04)	64 (0.01)	107 (0.02)	
**Presence of self-harm**				
No/not stated	173,431 (99)	687,559 (99.6)	860,990 (99.50)	< 0.001
Yes	1,770 (1.0)	2,589 (0.4)	4,359 (0.50)	
**Gestational diabetes**				
No/not stated	108,448 (96.5)	460,132 (94.7)	568,580 (94.99)	< 0.001
Yes	3,995 (3.6)	26,024 (5.4)	30,019 (5.01)	
**Gestational hypertension**				
No/not stated	45,501 (96.2)	206,355 (95.0)	251,856 (95.18)	< 0.001
Yes	1,803 (3.8)	10,952 (5.0)	12,755 (4.82)	
**Pre-eclampsia**				
No/not stated	45,986 (97.2)	211,944 (97.5)	257,930 (97.48)	< 0.001
Yes	1,318 (2.8)	5,363 (2.5)	6,681 (2.52)	

^A^ *p*-value was obtained from Student’s t-tests

^B^ *p*-value was obtained from a chi-square test

Table 1 also provides a comparison between partnered and non-partnered pregnant women, by demographic and health-related characteristics. In comparison to non-partnered women, partnered women were more likely to: be older; reside in a major city; have a lower level of socio-economic disadvantage; be a non-smoker; be born outside of Australia; be non-Indigenous; and/or have full insurance cover (all *p* < 0.001). In addition, partnered women, in comparison to non-partnered women were more likely to: have gestational diabetes; have gestational hypertension; not have preeclampsia; not have a depressive disorder; not have anxiety; and/or not self-harm (all *p* < 0.001).

Table 2 shows the association between marital status and mental disorders, after adjusting for the available confounders. The multiple logistic regression model estimated that the odds of depressive disorder was 2.75 times higher among the non-partnered pregnant women, compared with the partnered women (AOR = 2.75; 95%CI: 2.04, 3.70; *p* < 0.001). In addition, the odds of anxiety disorder during pregnancy was 3.16 times higher among non-partnered women (AOR: 3.16, 95%CI: 2.03, 4.91; *p* < 0.001). Furthermore, the model estimated that the odds of experiencing self-harm was two times higher among non-partnered pregnant women compared to their counterpart (AOR = 2.0; 95%CI: 1.82, 2.20; *p* < 0.001).

**Table 2:** Associations between marital status and mental disorders among admitted pregnant women in NSW, 2000–2011

Factor	Depressive disorder		Anxiety disorder		Self-harm	
	Model I	Model II	Model I	Model II	Model I	Model II^‡^
	OR (95% C.I.)	AOR (95% C.I.)	OR (95% C.I.)	AOR (95% C.I.)	OR (95% C.I.)	AOR (95% C.I.)
Marital status						
Partnered	1.00	1.00	1.00	1.00	1.00	1.00
Non-partnered	3.45 (2.68–4.45)	2.75 (2.04–3.70)^*^	2.91 (1.97–4.28)	3.16 (2.03–4.91)^*^	2.77 (2.56–3.00)	2.00 (1.82–2.20)^*^

**Model I:** Unadjusted model

**Model II:** Adjusted for sociodemographic factors (age, remoteness, socioeconomic disadvantage, country of birth and Indigenous status) and smoking status

**Model II**^**‡**^: Adjusted for sociodemographic factors (age, remoteness, socioeconomic disadvantage, country of birth and Indigenous status), smoking status, anxiety disorder and depressive disorder

Abbreviation: *AOR*, Adjusted Odds Ratio, *C.I* Confidence Interval

^*^p < 0.001

Hosmer and Lemeshow goodness-of-fit: for depressive disorder: χ2 = 8.37; *p* = 0.398, for anxiety disorder: χ2 = 4.73; *p* = 0.786; for self-harm: χ2 = 8.99; *p* = 0.343

## Discussion

This study identified several important findings regarding the relationship between marital status and prenatal mental disorders among admitted Australian women. In particular, non-partnered pregnant women were more likely to suffer from a depressive disorder, anxiety disorder or self-harm, than partnered pregnant women.

Our study found that prenatal depressive disorder is more likely among non-partnered pregnant women than their partnered counterparts. Various international epidemiological studies also found that non-partnered pregnant women (not married, single or not living together with a partner) have a higher odds of suffering from depressive symptoms during the prenatal period compared to partnered women [33, 43, 44, 57–60]. Furthermore, our study adds further evidence to the findings of previous research conducted in Victoria, Australia (*n* = 1578) [50] which found single mothers report higher levels of prenatal depressive symptoms compared to women with supportive partners. Interestingly, the authors also found that single pregnant women reported lower levels of depressive symptoms than those with unsupportive partners [50]. Some studies report a non-significant association between marital status and prenatal depression [46–48, 61]. The difference between the current and previous studies might be due to the variation in adjusting potential confounders and study participants' demographic characteristics. For example, a study conducted in UK adjusted for confounders such as marital satisfaction, previous history of mental illness and social support [47], and a study conducted in Italy also adjusted for confounders such as social support, stressful life events and relationship problem with a partner [46]. Furthermore, of the total participants (*n* = 546) of a study conducted in the US, most were single (91%, *n* = 497) [48], which can limit the statistical power of analysis and result in a non-significant association.

Our study demonstrated that the likelihood of prenatal anxiety disorder was three-fold higher among non-partnered women. Similarly, other studies conducted in Malaysia, Mexico, and Brazil have also shown that non-partnered pregnant women have a higher level of anxiety during the antenatal period [33, 59, 62] compared to partnered women. Conversely, a facility-based study conducted in China, Shanghai (*n* = 527) [49] reported a non-significant association between marital status and prenatal anxiety. The possible reason for the non-significant association could be that most of the study participants were married (98.6%, *n* = 520) [49], which can limit the statistical power of the analyses.

Our study also found that the likelihood of experiencing self-harm was two times higher among non-partnered pregnant women compared to those with partnered marital status. Support for the finding in the current study comes from cross-sectional studies conducted in Brazil (*n* = 1414) [45] and Ethiopia (*n* = 423) [63], focusing on pregnant women attending the antenatal care unit demonstrated that the likelihood of suicide was significantly related to lack of a cohabiting partner. A study conducted in Brazil [45] also performed a separate regression analysis for depressed pregnant women and found the odds of suicide was higher among non-partnered (single, divorced or widowed) depressed pregnant women (*n* = 315) [45]. Studies conducted in South Africa (*n* = 649) [64], Australia (*n* = 1507) [65] and US (*n* = 383) [66] reported a non-significant association between marital status and self-harm during pregnancy. The null result in a study conducted in South Africa could be due to adjusting for important confounders such as social support, marital stress, relationship with a partner, and previous history of mental illness, which was not possible in the current study [64]. Also, the study conducted in Australia which examined predictors of persistent self-harm (thought/attempt) adjusted for confounders such as intimate partner violence and afraid of partner during multivariate logistic regression analysis [65]. In a study conducted in US, Only 1 of the study participants attempted suicide during pregnancy, which might be due to the active participation of all study subjects in mental health treatment and willingly participated in the study, which might help in early identification and intervention of suicidal ideation [66]. Thus, the observed association in the current study could be due to inadequate adjustment of confounders. Further studies confirming this finding are recommended.

Almost 1 in 2500 pregnant women in the current study met the ICD-10-AM diagnostic criteria for depressive disorder. The prevalence in the present study is lower than the pooled prevalence of antenatal depression in high-income countries (17%) [4], as well as the reported prevalence of other studies conducted in Australia 7–17% [5, 6]. The discrepancy in the prevalence of prenatal depression might be due to the use of instruments to examine depression. For example, studies conducted in Australia used 10 item EPDS (10 items) with a score of ≥ 13, suggesting depressive symptoms, which are highly sensitive and inflate positive depression cases [67], whereas in the current study, depressed women were identified using a diagnostic tool (ICD-10-AM).

Our study also found that 1 in 5000 pregnant women diagnosed with anxiety disorder. A higher estimate of antenatal anxiety was reported from a study conducted in Australia (27%) [5] and other epidemiological studies (14–59%) [7–10]. The possible reason for the discrepancy could be that the study conducted in Australia used Beck Anxiety Inventory (BAI) (i.e., a score of ≥ 16) to screen anxiety symptoms, possibly inflating probable anxiety. In contrast, our study employed ICD-10 AM to diagnose a prenatal anxiety disorder.

Around 1 in 200 pregnant women in the current study diagnosed with intentional self-harm, which is lower than a report of an international review (5–14%) [68] and a study conducted in Australia (*n* = 1507) (5%) [65] and South Africa (*n* = 649) (18%) [64]. The discrepancy might be due to the fact that studies conducted in South Africa and Australia examined self-harm (thought/attempt) using a single-item screening tool from the Edinburgh Postnatal Depression Scale (EPDS) [69], which is more likely to inflate prenatal self-harm compared to ICD-10 AM.

Marital status [33, 43, 44] and the quality and length of the marriage relationship significantly affect prenatal mental health problems and the level of prenatal social support [38]. For pregnant women, a marital partner is one of the key sources of emotional and tangible support [70]. For many pregnant women, their marital partner plays a vital role in detecting perinatal depressive and anxiety symptoms and supports in seeking health care professional help [71, 72]. Also, partner support and marital stability are important protective factors for the psychological well-being of pregnant women [73]. Furthermore, partnered women have enormous psychosocial advantages compared to non-partnered women, though much of this may be restricted to women living in a quality marital relationship [74]. Despite the continued debate about the definition, the available evidence in developed countries viewed the quality of marital relationship as a multidimensional concept that measures objective features of marital relationship such as friendship, communication, affection, and trust, along with subjective features like marital satisfaction [75]. Studies have also shown that non-partnered pregnant women have a higher risk of antenatal depression and anxiety than women with a supportive partner [76, 77]. Also, an Australian population-based panel study found a decline in mental health for women who were separated or widowed [78]. Interestingly, partnered pregnant women living in poor-quality relationships with their spouses also appear at greater risk of prenatal anxiety and depression [38, 79, 80] because they are exposed to additional stress and anxiety from their spouses [51]. Besides, stress due to a challenging marital relationship with the spouse makes an adjustment to the current pregnancy difficult for the woman [81], subsequently leading to prenatal and postnatal mental health problem and adverse birth outcome [52]. A review of longitudinal studies examining maternal mental health in Australia and New Zealand indicated poor partner relationship as the strongest predictor of prenatal anxiety and depression [34]. Also, a study conducted in Canada (*n* = 3021) indicated that poor quality of marital relationships and partner tension significantly predicts prenatal anxiety [30]. Evidence also shown that lack of support from a partner coupled with barriers to health education correlates with decreased prenatal stress coping for low-income women [82]. Even though no study has yet examined the relationship between the mental well-being of the spouse and prenatal mental health problems, it could be a possible risk factor for prenatal mental health problems. Nonetheless, the psychopathology of the spouse played a significant positive role in the postnatal period for the occurrence of maternal depression [83].

### Strength and limitation

Our study has a number of important strengths. Our results are based on a large population-based administrative data linked from PDC and APDC sources. Also, it is the first study assessing the relationship between marital status and prenatal mental health problems (depressive disorder, anxiety disorder and self-harm) using high-quality linked data.

However, our study also has limitations. Since we did not have data on the following variables, our study did not adjust for the confounding role of social support, pre-pregnancy mental health problems, partner mental health status, intimate partner violence, length and quality of the marital relationship, which can play a key role in the observed associations. Evidence indicated the possible over-enumeration of admission due to depressive disorder, anxiety disorder, and self-harm might be because admissions could happen due to medical problems related to the perinatal period [84, 85]. During analysis, the overestimation of mental disorders was managed by including only admissions with a principal diagnosis of mental disorder. Lastly, more recent data should be examined to see if the associations identified in our analyses still hold.

## Conclusion

The partner status of Australian pregnant women has a significant positive association with prenatal depressive disorder, anxiety disorder and self-harm. This suggests it would be highly beneficial for maternal health care professionals to screen non-partnered pregnant women for prenatal mental health problems such as depression, anxiety and, self-harm. Also, screening non-pregnant women for social support is vital to assess the support level from other social networks. Policy-makers need to consider developing targeted community-based social support programs to enhance pregnant women's mental wellbeing.

## Supplementary Information

**Additional file 1.**

## Data Availability

All the available data are included in the manuscript.
